# Supplementary material for: Intervention provision and engagement in Colombia’s PAPSIVI – a national psychosocial support service for over half a million victims of armed conflict
Source: Confl Health. 2026 Feb 11;20:27. doi: 10.1186/s13031-026-00760-x (PMC12998117; doi:10.1186/s13031-026-00760-x)
Supplement: Supplementary file 1 — Supplementary Material 1 [file 13031_2026_760_MOESM1_ESM.docx]

Supplementary Material for

**Treatment assignment and treatment engagement in the world’s largest psychosocial support service for victims of armed conflict**

| *ICD Diagnostic Group* | *N* |
| --- | --- |
| Organic incl dementia (FO1-9) | 1070 |
| Psychoactive incl substance abuse (F10-19) | 906 |
| Schizophrenia (F20-29) | 1175 |
| Affective disorders (F30-39) | 4768 |
| Anxiety & stress-related (F40-48) | 8283 |
| Behavioural syndromes (F50-59) | 1927 |
| Adult personality disorders (F60-69) | 470 |
| Intellectual disabilities (F70-79) | 979 |
| Developmental disorders (F80-89) | 1829 |
| Disorders with childhood onset (F90-98) | 2311 |

**Table S1**. Numbers of attendees with prior mental health diagnosis by ICD diagnostic category

| *ICD Diagnostic Category* | *Individual* | *Group* | *Family* | *Community* |
| --- | --- | --- | --- | --- |
| Anxiety, stress-related (F40-48) | 1.21 (1.10 - 1.33) | 0.86 (0.45 - 1.63) | 0.88 (0.81 - 0.95) | 0.98 (0.89 - 1.08) |
| Affective disorders (F30-39) | 1.09 (0.98 - 1.21) | 0.30 (0.10 - 0.85) | 0.99 (0.90 - 1.08) | 0.92 (0.82 - 1.03) |
| Behavioural syndromes (F50-59) | 0.70 (0.59 - 0.83) | 0.55 (0.15 - 2.07) | 1.06 (0.93 - 1.20) | 1.44 (1.24 - 1.66) |
| Organic incl dementia (FO1-9) | 0.88 (0.71 - 1.08) | 2.47 (0.76 - 8.05) | 1.29 (1.06 - 1.56) | 0.71 (0.56 - 0.90) |
| Childhood onset (F90-98) | 0.75 (0.62 - 0.90) | 1.00 (0.49 - 2.02) | 1.05 (0.91 - 1.21) | 1.28 (1.05 - 1.55) |
| Developmental disorders (F80-89) | 0.85 (0.70 - 1.02) | 2.42 (1.29 - 4.54) | 1.09 (0.94 - 1.27) | 0.86 (0.69 - 1.07) |
| Intellectual disabilities (F70-79) | 0.54 (0.41 - 0.70) | 0.97 (0.22 - 4.23) | 1.57 (1.27 - 1.93) | 0.95 (0.73 - 1.25) |
| Personality disorders (F60-69) | 1.09 (0.82 - 1.45) | 1.11 (0.27 - 4.61) | 0.95 (0.73 - 1.22) | 0.99 (0.71 - 1.39) |
| Psychoactive/substance (F10-19) | 0.95 (0.74 - 1.21) | 0.06 (0.01 - 0.41) | 1.24 (1.01 - 1.52) | 0.73 (0.54 - 0.99) |
| Schizophrenia / psychotic (F20-29) | 1.07 (0.85 - 1.33) | 0.87 (0.20 - 3.73) | 0.91 (0.76 - 1.09) | 1.11 (0.91 - 1.36) |

**Table S2**. Associations (odds ratio and 95% confidence intervals) between ICD diagnostic category and treatment modality assignment
